# Prostate cancer stroma: an important factor in cancer growth and progression

**DOI:** 10.17305/bjbms.2015.449

**Published:** 2015-05

**Authors:** Božo Krušlin, Monika Ulamec, Davor Tomas

**Affiliations:** Department of Pathology, Sestre milosrdnice University Hospital and School of Medicine, University of Zagreb, Zagreb, Croatia

**Keywords:** Prostate cancer, stroma, myofibroblast, extracellular matrix components

## Abstract

Reactive stromal changes that occur in different human cancers might play a role in local tumor spreading and progression. Studies done on various human cancers have shown activated stromal cell phenotypes, modified extracellular matrix (ECM) composition, and increased microvessel density. Furthermore, they exhibit biological markers consistent with stroma at the site of wound repair. In prostate cancer, stroma is composed of fibroblasts, myofibroblasts, endothelial cells and immune cells. Predominant cells in the tumorous stroma are, however, fibroblasts/myofibroblasts. They are responsible for the synthesis, deposition and remodeling of the ECM. Epithelial tumorous cells, in interaction with stromal cells and with the help of various molecules of ECM, create a microenvironment suitable for cancer cell proliferation, movement, and differentiation. In this review, we discussed the role of different stromal components in prostate cancer as well as their potential prognostic and therapeutic significance.

## INTRODUCTION

Prostatic cancer is the most common malignancy in men and the second cause of cancer death in developed countries.

The prostatic gland is composed of two distinctive compartments: epithelial and stromal. They mutually interact via androgen receptors and this interplay is important for prostate development and differentiation [1-3]. Analogously, prostate cancer is composed of both malignant epithelial cells and supportive stroma whose transformation is important for the growth and development of the tumor. Cancerous stroma is composed of fibroblasts, myofibroblasts, endothelial cells and immune cells. Predominant cellular types are, however, fibroblasts or myofibroblasts, which play an important role in synthesis, deposition and remodeling of the extracellular matrix. Tumorous epithelial cells, in interaction with stromal cells, and with the help of various molecules of extracellular matrix (ECM) create a microenvironment suitable for cancer cell proliferation, movement, and differentiation [4-13].

Reactive stromal changes occurring in different human cancers might play a role in local tumor spread and progression. Studies on different human cancer specimens have demonstrated activated stromal cell phenotypes, modified ECM composition, and increased microvessel density. Furthermore, they exhibit biological markers consistent with stroma at the site of wound repair [1, 5, 7, 12,13].

Tumor cell populations have several important features: capacity for self-renewal, ability to survive under different stress conditions and potential to produce metastases, the latter resulting in increased cancer aggressiveness and widespread dissemination. It appears that not only all the cells of tumor stroma, but also the other stromal components can potentially affect tumorigenesis. They play a key role in enhancement of tumor progression by stimulating angiogenesis and promoting cancer cell survival, proliferation, and invasion. In epithelial-stromal transformation, a panel of highly motile, independent cells capable of invasion and metastasis is involved [5-15].

Presently, it is evident that both malignant transformation and tumor progression are not exclusively regulated by disruption of oncogenes and tumor suppressor genes in neoplastic cells. Other factors, such as the modified interaction between stromal and epithelial compartments that influence androgen receptors, and studies on molecular pathways, signal molecules, and molecules of ECM involved in prostate carcinogenesis are crucial to the better understanding of cancer development and progression [1-14].

Different models were used to study cell to cell and ECM interactions in prostate cancer: cell line cultures, animal models, and prostate cancer tissue specimens obtained at intraoperative consultations and tissue processed and embedded in paraffin blocks. Preclinical animal models on rats, mice and dogs were established in an attempt to mimic the initial steps of prostate carcinogenesis as well as carcinomatous progression and metastatic potential [14-17].

## ANDROGEN RECEPTORS

Androgen plays a significant role in the development of the prostate gland, whereas stromal cells are crucial to maintaining its proper function. In order to become differentiated, prostatic epithelial cells require the presence of androgen receptors both in stromal and epithelial parts of prostate [2-4,8].

It is well known that this interaction performed via androgen receptors (AR) is also important in prostate carcinogenesis. Epithelial AR deprivation therapy is used in prostate cancer, but it cannot completely suppress the growth of the tumor. Stromal AR, thus, appear to have a more important role than AR in epithelial tumorous cells. Activated stromal AR affect stromal myofibroblasts, through which prostate carcinoma progression is promoted, and seem to be significant even in androgen-resistant tumors [18-21]. These stromal receptors may be possible targets for future anticancer therapies and are the subject of many studies done on in vitro cell lines, tissue recombination experiments, and androgen receptor knockout animal models.

## FIBROBLASTS AND MYOFIBROBLASTS

Myofibroblasts are dynamic stromal cells found at the site of pathologic tissue remodeling. Carcinoma cells have the ability to transform fibroblasts into reactive myofibroblasts, which synthesize different ECM components: collagen, fibronectin, tenascin, versican, galectin, laminin and others. Myofibroblasts can also express proteases and secrete growth factors that support angiogenesis. They are crucial cells that create a tumor-promoting reactive stroma setting, and can stimulate cancer cell growth and migration [22-24].

Prostate cancer-reactive stroma is composed of a myofibroblasts and fibroblasts mixture, with a significant decrease in fully differentiated smooth muscle, whereas normal prostate stroma consists predominantly of smooth muscle [25-27]. Proteins of the ECM play role in cell adhesion and cell signaling, and remodeling of ECM influences cancer spread and invasion. The “new” microenvironment, created in this way, is continuously changing to support the formation of glandular structures and tumor structures, as shown in some animal models [21, 27-29].

The origin of carcinoma fibroblasts and myofibroblasts, including the origin of stromal fibroblasts and bone marrow-derived mesenchymal stem cells, remains questionable, so as the epithelial-endothelial-mesenchymal transition process [9,12,14]. Mechanical force has been studied as one of the factors in conversion of fibroblasts into myofibroblasts. It was investigated with different cell types employing microfluid platforms. The expansion of tumor cells induces mechanical changes in multiple fibroblastic populations in the tumor microenvironment, exerting force on surrounding tissue and inducing local compressive stress [30].

**Figure 1:**
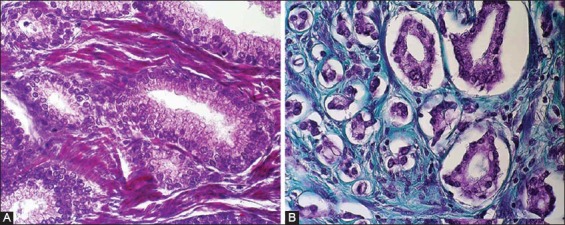
Mallory or Masson trichrome staining in A) benign prostate hyperplasia (x400) and B) prostate cancer (x400).

Fibroblast and myofibroblastic stromal changes in prostate carcinoma could be easily quantified by a simple histochemical method (Mallory or Masson trichrome staining) (Figure 1) as well as by an immunohistochemical procedure using antibodies to vimentin, α- smooth muscle actin and desmin [5,25]. Loss of the smooth-muscle cells, quantified immunohistochemically by intensity of the stromal changes, and the appearance of the stromal fibro- and myofibroblasts was associated with a shorter disease-free period and the worse outcome [31,32].

A relatively recently described phenomenon, that might be related to the fibro- or myofibroblasts and their products, is the appearance of the so-called periacinar halos, retraction clefting or cleft-like spaces within a neoplastic prostatic tissue. The neoplastic cells of prostatic cancer often appear pulled away from the surrounding stroma, leaving halos around the acini [33-36]. Some authors have suggested that this retraction artifact might serve as an additional criterion in the diagnosis of carcinoma, especially when prominent and identified in the half or more of the gland [33-37]. The most pronounced periacinar retraction was noticed in the association with Gleason pattern 3 adenocarcinoma. Some authors have suggested that this phenomenon is probably only an artifact [38]. Conversely, other authors attributed the periacinar retraction to the stromal changes present in prostatic adenocarcinoma and did not consider these clefts to be a simple artifact. Similarly to the retraction artifact, it has been shown that the stromal reaction in prostatic carcinoma is more pronounced in Gleason pattern 3 [25,39]. Furthermore, periacinar retraction artifact was also proposed as an additional and helpful diagnostic criterion in breast and urothelial carcinoma [40,41]. In addition, recent results revealed that the retraction artefact in prostatic carcinoma correlates with different clinicopathological features of the tumor as well as with the biochemical recurrence-free survival, pointing out that the presence and the extent of the retraction artifact could predict worse outcome in patients with prostatic adenocarcinoma [42]. The similar prognostic significance of retraction artifact has been reported in breast carcinoma [43,44].

## COLLAGENS

Collagens are fibrillar proteins that form a three-dimensional frame of ECM and are important in the cell signaling processes and metabolism. They are a fundamental part of stromal changes that affect tumor progression, cell survival, apoptosis and cell invasion [9]. Collagens are produced by fibroblasts and myofibroblasts. In prostate cancer, the network of collagen fibers is loose, and its organization is disturbed. Metabolic changes in carcinomatous stroma are increased compared to metabolic changes present in normal prostate stroma. Type I collagen is thorn and results in formation of biologically active collagen I peptides, which then facilitate proliferation and angiogenesis. Also, collagen type I can induce a reduction of E-cadherin-mediated cell–cell adhesion and the loss of E-cadherin, which is important for invasion capability. Type I collagen slitting is also required for angiogenesis at tumor sites. Decreased density of collagen and reticular fibers were found in human prostate tumor stroma [45-47]. Therefore, the assessment of changes in fibrillar components that affect the stromal environment in prostate cancer may help in the evaluation of tumor aggressiveness [29].

## ELASTIC FIBERS

Elastic fibers are important for tissue flexibility. Similar to collagens, after elastin degradation, elastin peptides induce stromal cells (fibroblasts, macrophages, lymphocytes, smooth muscle cells and endothelial cells) via the elastin–laminin receptor. There are limited data on the role of elastin and its receptors in tumor invasion, but they are disorganized in the stroma of prostate cancer.

Elastin and its peptides are factors involved in tumor invasion, because these molecules are known to stimulate receptor signaling and chemotaxis. This could explain the morphometric changes reported in certain tumor cell lines invading elastic lamina [29, 46].

## LAMININS

**Figure 2:**
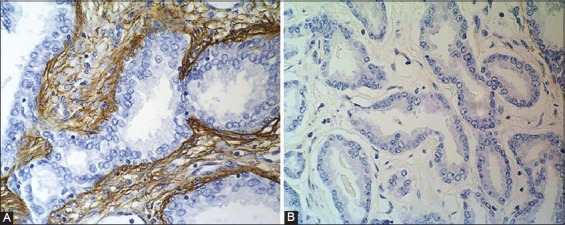
Immunohistochemical laminin staining in A) benign prostate hyperplasia, showing positive cells in stroma (x400) and B) prostate cancer, showing negative cells in stroma (x400).

Laminins are heterotrimeric molecules made up by one α, one β and one γ chain. Thus far, five α-chains, three β-chains and three γ-chains have been described. These chains combine into at least 14 different types of laminin. The distribution of these laminin isoforms varies between tissues, but in most basal membranes (BMs) more than one type of laminin is present. Laminins are associated with cell differentiation, preservation of cell shape and movement, maintenance of tissue phenotypes, promotion of tissue survival and are present in the basal lamina. Their functions in tumor invasion are the subject of extensive research [48-50]. Some studies reported significant decrease in the expression of laminin in carcinoma comparing to the adjacent prostate tissue (Figure 2) [39]. It has also been shown that membrane type 1 matrix metalloproteases are modifying the laminin-rich basal membrane, playing thus a role in transformation of prostate intraepithelial neoplasm into invasive cancer through their capacity to degrade laminin [39, 51,52].

## TENASCIN-C

Tenascin-C is a large (180–300 kDa), hexameric multidomain glycoprotein and located mainly in the ECM. It is involved in tissue interactions during embryogenesis, wound healing, inflammation, and oncogenesis. Tenascin-C is considered to be an anti-adhesive molecule in the stroma, modulating adhesion between cells via fibronectin, an important factor in the cell to ECM adhesion. Tenascin expression in prostatic tissue is considered to decrease or disappear after the maturation of the gland is finished. Some studies reported that tenascin expression is increased in prostate cancer stroma and tenascin-C significantly increased in stroma around neoplastic glands. These data suggest a potential role of tenascin-C in the regulation of tumor cell proliferation, invasion, and metastasis [52-57].

**Figure 3:**
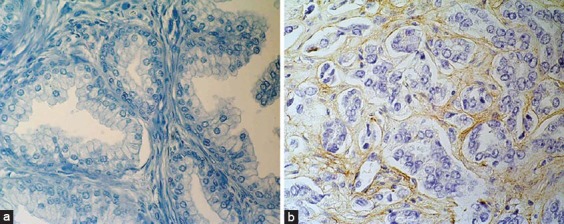
Immunohistochemical tenascin-C staining in A) benign prostate hyperplasia, showing negative cells in stroma (x400) and B) prostate cancer, showing positive cells in stroma (x400).

In our experience, the expression of tenascin-C was significantly increased in carcinomatous tissue compared to the adjacent peritumourous tissue and BPH. Tenascin-C was predominantly expressed in stroma around neoplastic glands but was also expressed in the wall of medium-sized blood vessels, which served as an internal positive control. However, in only a few cases we noted a weak positive tenascin reaction in the cytoplasm of neoplastic epithelial cells (Figure 3) [39].

## GALECTIN-3

Galectin-3 interacts with the intracellular glycoproteins, cell surface molecules, and the extracellular matrix proteins. According to the present data, it is down-regulated in prostate cancer. Van den Brule et al. suggested that galectin-3 might play an anti-tumor role when present in the nucleus, whereas it could favor tumor progression when expressed in the cytoplasm of the tumorous epithelial cells [58]. Other authors did not confirm these results, but it was suggested that the expression of galectin-3 in cytoplasm of the epithelial cells correlates positively with tumor progression [59]. It is shown that galectin-3 can inhibit anticancer drug-induced apoptosis through regulation of Bad protein and suppression of the mitochondrial apoptosis pathway [60]. It could be one of the target proteins for cancer treatment. The presence of galectin-3 in the stroma, however, indicated an unfavorable prognosis [58-62].

**Figure 4:**
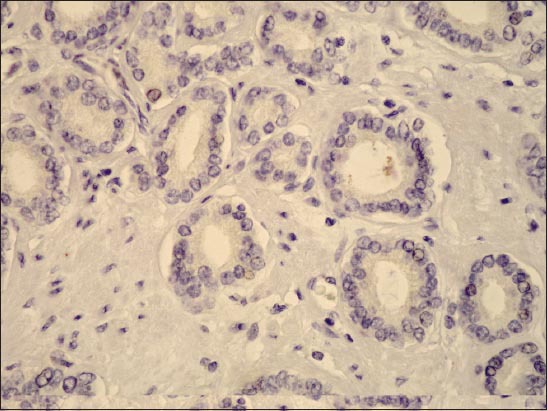
Immunohistochemical galectin-3 staining in prostate cancer, showing negative cells in stroma (x400).

In our experience, the expression of galectin-3 was significantly decreased in carcinoma compared to adjacent peritumourous tissue and BPH (Figure 4).

## CONNEXINS

Connexins are transmembrane proteins that form intercellular channels important for cell communication and adhesion. It is also a tumor suppressor gene (protein) [63-65]. Dysfunction of connexins plays a role in prostate carcinogenesis and its expression is often reduced during tumor progression and metastasis. In study of Benko et al. decreased connexin 43 (Cx43) expression was related to the prostate cancer progression. Lower Cx43 expression was associated with shorter follow-up time, indicating a shorter disease-free survival and higher preoperative PSA values [66].

## SYNDECAN-2

Syndecan-2 (SDC2) is a heparan-sulfate glycosaminoglycan, which participates in cell adhesion and migration, and is known to play a role in cancer progression and neoangiogenesis [67,68]. SDC2 overexpression in prostate cancer was significantly associated with the established features indicative of worse prognosis, such as a higher preoperative PSA value, a higher Gleason score, positive surgical margins and the extraprostatic extension. Expression of SDC2 was also associated with the biochemical disease progression [69].

## METALLOPROTEINASES

An elevated levels of metalloproteinases were registered in the stroma of various cancers. Matrix metalloproteinases (MMP) are produced by both tumor epithelial and stromal cells. MMPs are able to degrade a variety of ECM molecules, and regulate signaling pathways that control cell growth, survival, invasion, inflammation, and angiogenesis. In normal tissue MMP activity is carefully controlled, but in cancer their control mechanisms are altered. In a recently published study, it was found that expression of MMP-2, MMP-3, and MMP-9 was increased, favoring tumor progression [51,52].

## ANGIOGENESIS

Angiogenesis is important for tissue growth as well as for tumor generation, progression, and its malignant behavior. Neovascularization in prostate carcinoma develops not only through cell-to-cell interaction, but also via multiple autocrine, paracrine and mechanical factors, and is similar to the one seen in the wound repair process [8-12]. As previously mentioned, prostate cancer stroma is composed of ‘reactive’ fibroblasts and myofibroblasts, responsible for extracellular matrix remodeling and increase in local vascular density. Stromal cells are capable of regulating angiogenesis by various protein and cytokine molecules. Protein ps20 is known to enhance endothelial cell motility and its synthesis is stimulated by TGF-β [70]. Another humoral factor, vascular endothelial growth factor (VEGF), is also reported to act as an endothelial cell mitogen and can be synthesized by both epithelial cells and myofibroblasts. Some studies confirmed that VEGF expression in prostate cancer correlates to a PSA level and Gleason score [71, 72].

## GROWTH FACTORS, PEPTIDES AND RECEPTORS

Prostate cancer stromal cells express epidermal growth factor (EGF) as well as transforming growth factor-α (TGF-α). These factors are also synthesized by malignant epithelial cells and signal through epidermal growth factor receptors (EGFR). Autocrine expression of EGF and TGF-α affects the autonomous growth of human prostate cancer. Also, it seems that EGF plays an important role in stimulation of invasiveness of prostate cancer by promoting chemomigration of tumorous cells. The EGFR family-related oncogenes HER-2/neu, HER-3, and HER-4 are also differentially expressed in the stroma of prostate cancer. HER-4 receptor protein is strongly expressed in normal epithelial cells, but not in cancer.

Transforming growth factor-β (TGF-β) increases cancer growth and metastasis because of the altered expression of TGF-β receptors. This signaling pathway is downregulated in prostate cancer. The expression of TGF-β RI and RII proteins is reduced in both the primary cancer and lymph node metastases [73,74].

Human cancer cells acquire autocrine expression of fibroblasts growth factor-2 (FGF-2), which encourage cancer cell proliferation and elevates the titer of FGF-2 in patients’ serum. FGF-2 regulates changes in ECM by modulating expression of proteases and promoting the synthesis of collagen, fibronectin, and proteoglycans.

Prostatic stromal cells secrete insulin-like growth factor-I and II (IGF-I, II), which stimulate the growth of epithelial tumor cells via the EGFR signal transduction cascade. However, its importance in prostate carcinogenesis is still unclear.

It appears that nerve growth factor (NGF) is produced by both stromal and epithelial tumor cells, but prostate cancer cells that produce autocrine NGF are able to escape paracrine dependence of stromal cell-derived NGF. Upregulation of autocrine neurotrophin expression may be associated with invasion along the perineural space and metastasis [75].

Vascular endothelial growth factor (VEGF) promotes angiogenesis in prostate carcinoma.

Platelet-derived growth factors (PDGF) contribute to cell proliferation, survival, transformation, and chemotaxis. Prostate cancer expresses both PDGF-A and the PDGF-A receptor, which are presumed to play a role in malignant transformation in prostate cancer. PDGF-B has not been detected in this type of carcinoma [74-77].

## CYTOKINES AND RECEPTORS

Hepatocyte growth factor (HGF) is expressed in the stroma of the human prostate. It stimulates proliferation and motility of cancer cells, interacting through c-met protooncogene product located in the epithelial cells.

Other cytokines, such as interleukins -1 (IL-1), -2 (IL-2), or interferon-alpha, -beta, and -gamma are also expressed during prostatic carcinogenesis. Studies have reported contradictory results about IL-6 signaling and IL-6 receptors in cancer cells in vitro and in tissue. IL-10 upregulates expression of tissue inhibitor of metalloproteinase-1, -2 and -9 which is consistent with its overall inhibitory effect on cancer cells [78].

## CONCLUSIONS

Many mechanisms are involved in the biology of prostate cancer growth and progression. Collaboration between epithelial and stromal compartments, that both interact under the influence of androgen and other hormonal factors, is among the most important, resulting in a formation of microenvironment suitable for cancer growth and progression. Consequently, the interaction of different extracellular matrix proteins, glycoproteins, metalloproteinases, growth factors and their receptors is altered. A new suitable environment is created for neovascularization and survival of resistant cell clones capable of self-renewal, invasion and metastasis. In addition, these stromal changes could serve as valuable additional tools in diagnosis and prognosis of prostate cancer. Further investigation is needed for the novel therapeutic possibilities that could influence stromal cells in the tumor.
